# International survey on influenza-associated pulmonary aspergillosis (IAPA) in intensive care units: responses suggest low awareness and potential underdiagnosis outside Europe

**DOI:** 10.1186/s13054-020-2808-8

**Published:** 2020-03-11

**Authors:** Karin Thevissen, Cato Jacobs, Michelle Holtappels, Mitsuru Toda, Paul Verweij, Joost Wauters

**Affiliations:** 1 KU Leuven Centre of Microbial and Plant Genetics, Leuven, Belgium; 2 KU Leuven Department of Microbiology, Immunology and Transplantation, Laboratory for Clinical Infectious and Inflammatory Disorders, Herestraat 49, 3000 Leuven, Belgium; 3 Mycotic Diseases Branch, Centers for Disease Control and Prevention, Atlanta, GA USA; 4 Department of Medical Microbiology, Radboudumc Center of Infectious Diseases (RCI) Netherlands; Centre of Expertise in Mycology, Radboudumc/CWZ, Nijmegen, the Netherlands

Dear Editor,

Historically, fungal infections have not been considered an important influenza complication. In 2018, a retrospective multicenter cohort study in Belgium and the Netherlands identified aspergillosis in 19% of patients with severe influenza. As influenza seemed independently associated with IPA, the term influenza-associated pulmonary aspergillosis (IAPA) was introduced [1, 2]. In contrast, a single-center retrospective Canadian study reported an incidence of 7.2% [3]. Incidence seemingly varies between geographical regions and centers, but awareness among physicians may also vary. Diagnosis of IAPA is still challenging. Since culture has low sensitivity, non-culture-based diagnostic methods like galactomannan (GM) should be used [4].

As no data exist on IAPA awareness in different parts of the world, nor on differences in clinical use of GM in broncho-alveolar lavage (BAL) or serum in critically ill influenza patients, we designed a simple survey (Table 1) and invited 20,093 members of the ELSO, SCCM, and ESICM to participate. A total of 565 responses were received, of which 90% from critical care physicians. Notably, 40% respondents were based in the US, 37% in Europe, and 22% in other continents (Fig. 1a).

**Table 1:** Overview of respondent’s input based on the survey

	Responses			
	Total	Europe	U.S.	Other^a^
**Valid respondents**	565 (100%)	208 (37%)	224 (40%)	133 (23%)
**Role at ICU**				
Critical care physician	509/565 (90%)	197/208 (95%)	186/224 (83%)	126/133 (95%)
Infectious diseases physician	8/565 (1%)	4/208 (2%)	3/224 (1%)	1/133 (0.5%)
Nurse	9/565 (2%)	1/208 (1%)	7/224 (3%)	1/133 (0.5%)
Other	39/565 (7%)	6/208 (2%)	28/224 (13%)	5/133 (4%)
**Number of ICU beds**				
< 20 beds	176/554 (32%)	94/207 (46%)	27/222 (12%)	55/125 (44%)
21–60 beds	226/554 (41%)	85/207 (41%)	89/222 (40%)	52/125 (42%)
61–100 beds	68/554 (12%)	17/207 (8%)	43/222 (19%)	8/125 (6%)
> 100 beds	84/554 (15%)	11/207 (5%)	63/222 (29%)	10/125 (8%)
**Number of severe influenza cases per season**				
< 10 cases	132/557 (23%)	56/206 (27%)	32/222 (14%)	44/129 (34%)
11–30 cases	272/557 (49%)	118/206 (57%)	99/222 (45%)	55/129 (43%)
31–50 cases	60/557 (11%)	18/206 (9%)	30/222 (14%)	12/129 (9%)
> 50 cases	49/557 (9%)	10/206 (5%)	27/222 (12%)	12/129 (9%)
I do not know	44/557 (8%)	4/206 (2%)	34/222 (15%)	6/129 (5%)
**NAIs as standardized treatment**				
Yes	416/556 (75%)	162/206 (79%)	165/222 (74%)	89/128 (70%)
Yes, but only if influenza symptoms started ≤ 48–72 h before ICU admission	97/556 (17%)	34/206 (17%)	41/222 (19%)	22/128 (17%)
No	27/556 (5%)	7/206 (3%)	3/222 (1%)	17/128 (13%)
I do not know	16/556 (3%)	3/206 (1%)	13/222 (6%)	0
**Obtaining lower respiratory samples**				
Always	78/554 (14%)	52/205 (25%)	10/220 (5%)	16/129 (12%)
Very often	139/554 (25%)	67/205 (33%)	43/220 (19%)	29/129 (22%)
Sometimes	187/554 (34%)	50/205 (24%)	97/220 (44%)	40/129 (31%)
Rarely	129/554 (23%)	31/205 (15%)	65/220 (29%)	33/129 (26%)
Never	16/554 (3%)	5/205 (3%)	1/220 (1%)	10/129 (8%)
N/A—have not treated patients	5/554 (1%)	0	4/220 (2%)	1/129 (1%)
**Galactomannan testing in BAL**				
Always	52/551 (9%)	38/204 (19%)	5/220 (2%)	9/127 (7%)
Very often	65/551 (12%)	38/204 (19%)	14/220 (6%)	13/127 (10%)
Sometimes	107/551 (19%)	37/204 (18%)	46/220 (21%)	24/127 (19%)
Rarely	163/551 (30%)	43/204 (21%)	83/220 (38%)	37/127 (29%)
Never	143/551 (26%)	44/204 (21%)	61/220 (28%)	38/127 (30%)
N/A—have not treated patients	21/551 (4%)	4/204 (2%)	11/220 (5%)	6/127 (5%)
**Galactomannan testing in serum**				
Always	39/554 (7%)	28/205 (14%)	5/220 (2%)	6/129 (5%)
Very often	60/554 (11%)	36/205 (18%)	11/220 (5%)	13/129 (10%)
Sometimes	115/554 (21%)	42/205 (20%)	46/220 (21%)	27/129 (21%)
Rarely	175/554 (31%)	47/205 (23%)	94/220 (43%)	34/129 (26%)
Never	142/554 (26%)	48/205 (23%)	51/220 (23%)	43/129 (33%)
N/A—have not treated patients	23/554 (4%)	4/205 (2%)	13/220 (6%)	6/129 (5%)
**Number of IAPA in influenza patients in the past 5 years**				
No	347/553 (63%)	85/204 (41%)	183/220 (83%)	79/129 (61%)
Yes, 1 patient	77/553 (14%)	34/204 (17%)	21/220 (9%)	22/129 (17%)
Yes, 2–5 patients	99/553 (18%)	61/204 (30%)	15/220 (7%)	23/129 (18%)
Yes, > 5 patients	30/553 (5%)	24/204 (12%)	1/220 (1%)	5/129 (4%)

Descriptive statistics were used to analyze the differences in proportions of responses between Europe, the US, and other countries. Fisher’s exact or *χ*^2^ test was used to calculate the *p* values. Correction for multiple comparisons was applied. The Spearman rank-order correlation coefficient was used to determine univariate correlations between parameters. A *p* value of < 0.05 was considered statistically significant. Results were analyzed using SPSS (IBM SPSS Statistics version 26). *ICU* intensive care unit, *N/A* not applicable, *BAL* bronchoalveolar lavage, *IAPA* influenza-associated pulmonary aspergillosis

^a^Other countries + unknown

**Fig. 1:**
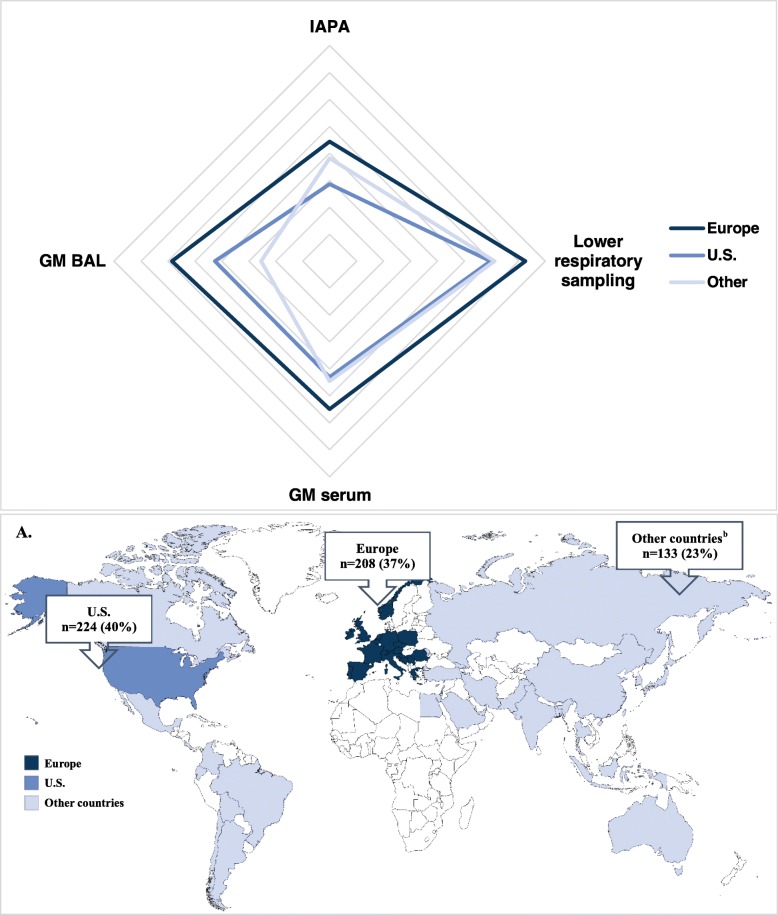
Number of respondents and their geographical location and web diagram representing the mean response for Europe, United States and other countries. **a**^a^Austria, Belgium, Bosnia and Herzegovina, Croatia, Czech Republic, Denmark, France, Germany, Greece, Ireland, Italy, Netherlands, Norway, Poland, Portugal, Romania, Serbia, Slovenia, Spain, Switzerland, United Kingdom; ^b^Argentina, Australia, Bangladesh, Barbados, Bolivia, Brazil, Canada, Chile, China, Colombia, Costa Rica, Ecuador, Egypt, El Salvador, Georgia, India, Indonesia, Iran, Israel, Japan, Lebanon, Malaysia, Mexico, Moldova, Pakistan, Palestine, Philippines, Russia, Saudi Arabia, South Korea, Taiwan, Thailand, Tunisia, Turkey, United Arab Emirates **b** Mean responses were calculated based on histograms. Subdivisions represent 0.5 arbitrary units for each of the correlation variables. For the GM BAL, GM serum and lower respiratory sampling variables, we used following units: 1 combining the categories ‘never’ and ‘rarely’; 2: sometimes; and 3 combining the categories ‘very often’ and ‘always’

The majority (72%, *n* = 404) of respondents reported up to 30 severe influenza cases per season. Globally, 63% (*n* = 347) of respondents had never heard of or seen IAPA in the past 5 years. In contrast to the US (17%, *n* = 37) and other countries (39%, *n* = 50), a majority of European participants (58%, *n* = 119) was familiar with IAPA.

Less than half of respondents (39%, *n* = 217) indicated frequent sampling of lower respiratory specimens, whereas 26% (*n* = 145) rarely or never performed sampling. We observed differences across different countries: European respondents performed lower respiratory sampling very often or always (58%, *n* = 119). This was more than the respondents in the US (24%, *n* = 53; *p* < 0.001) or those in other countries (33%, *n* = 45; *p* < 0.001).

While 39% of respondents did take lower respiratory samples, the majority of respondents (79%, *n* = 434) seldom determined GM in BAL. In general, GM determination in BAL/serum was more frequently reported by respondents in Europe than in the US (*p* < 0.01) or other countries (*p* < 0.01). Interestingly, both GM determination in BAL and serum correlated with the reported number of IAPA cases in all regions. Based on the calculated mean of response histograms, a web diagram was constructed, showing that a higher number of observed IAPA cases were associated with more intensive sampling (Fig. 1b).

Our results show that differences exist in awareness and diagnostic practices related to IAPA among surveyed ICU clinicians in Europe, the US, and other countries. Moreover, many clinicians were unaware of the association between influenza and aspergillosis, with European respondents having seen or heard more frequently of IAPA cases than those in the US and other countries. Although the observed differences in IAPA cases could be explained by true variation in IAPA prevalence (e.g., due to differences in environmental/genetic factors, influenza vaccination coverage, use of antiviral therapy or steroids [5, 6]), the condition might be underdiagnosed outside Europe, which is supported by lower use of GM testing on BAL or serum. Of course, these findings might not necessarily be generalizable due to the low response rate (3%). Actually, the questions were deliberately kept simple and straightforward to increase the response rate. Anyway, greater awareness of IAPA is needed as are rapid diagnostic tests. Based on the conclusions of this survey, it is clear that more multicentric prospective studies are needed to assess the incidence and risk factors for IAPA in different parts of the world, thereby taking the most updated guidelines on diagnostic and sampling practices into account, as well as the use of steroids and the consensus definitions regarding fungal infection versus colonization.

## Data Availability

All data generated or analyzed during this study are included in this published article.

## Abbreviations

IAPA: Influenza-associated pulmonary aspergillosis
IPA: Invasive pulmonary aspergillosis
GM: Galactomannan
BAL: Broncho-alveolar lavage
ELSO: Extracorporeal Life Support Organization
SCCM: Society of Critical Care Medicine
ESICM: European Society of Intensive Medicine
US: United States
ICU: Intensive care unit
