# Correction to: International survey on influenza-associated pulmonary aspergillosis (IAPA) in intensive care units: responses suggest low awareness and potential underdiagnosis outside Europe

**DOI:** 10.1186/s13054-020-02901-x

**Published:** 2020-04-29

**Authors:** Karin Thevissen, Cato Jacobs, Michelle Holtappels, Mitsuru Toda, Paul Verweij, Joost Wauters

**Affiliations:** 1 KU Leuven Centre of Microbial and Plant Genetics, Leuven, Belgium; 2 KU Leuven Department of Microbiology, Immunology and Transplantation, Laboratory for Clinical Infectious and Inflammatory Disorders, Herestraat 49, 3000 Leuven, Belgium; 3 Mycotic Diseases Branch, Centers for Disease Control and Prevention, Atlanta, GA USA; 4 Department of Medical Microbiology, Radboudumc Center of Infectious Diseases (RCI) Netherlands; Centre of Expertise in Mycology, Radboudumc/CWZ, Nijmegen, the Netherlands

**Correction to: Crit Care 24, 84 (2020)**

**https://doi.org/10.1186/s13054-020-2808-8**

In the publication of this article [1], there was an error in Fig. 1 which caused that the a, b were switched and ‘b’ was missing as a caption on Fig. 1b. This has now been updated in the original article.

The correct figure and caption is:

**Fig. 1:**
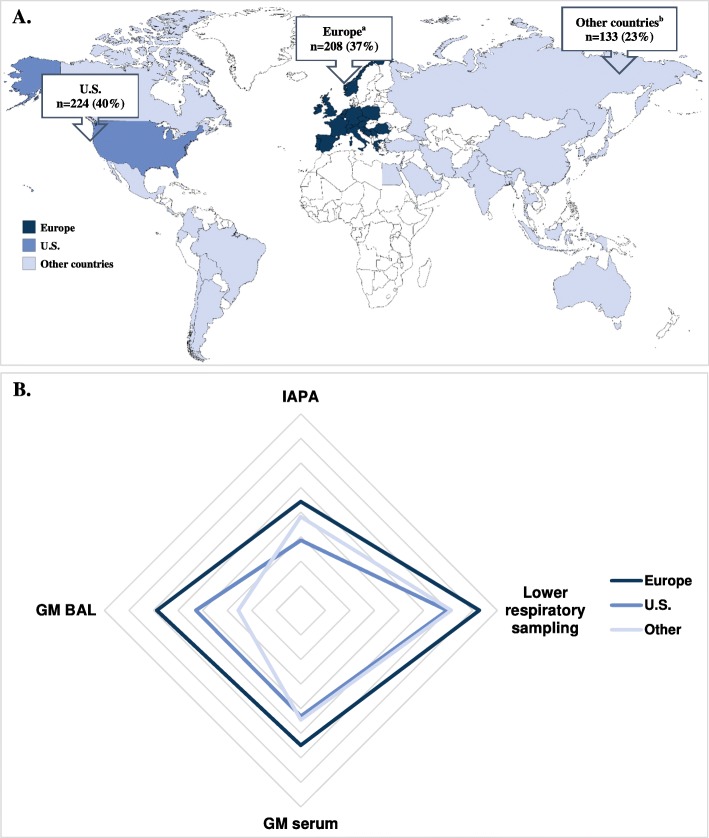
Number of respondents and their geographical location and web diagram representing the mean response for Europe, the US, and other countries. **a**^a^Austria, Belgium, Bosnia and Herzegovina, Croatia, Czech Republic, Denmark, France, Germany, Greece, Ireland, Italy, Netherlands, Norway, Poland, Portugal, Romania, Serbia, Slovenia, Spain, Switzerland, and UK; bArgentina, Australia, Bangladesh, Barbados, Bolivia, Brazil, Canada, Chile, China, Colombia, Costa Rica, Ecuador, Egypt, El Salvador, Georgia, India, Indonesia, Iran, Israel, Japan, Lebanon, Malaysia, Mexico, Moldova, Pakistan, Palestine, Philippines, Russia, Saudi Arabia, South Korea, Taiwan, Thailand, Tunisia, Turkey, and United Arab Emirates. **b** Mean responses were calculated based on histograms. Subdivisions represent 0.5 arbitrary units for each of the correlation variables. For the GM BAL, GM serum, and lower respiratory sampling variables, we used the following units: (1) combining the categories “never” and “rarely,” (2) sometimes, and (3) combining the categories “very often” and “always”
